# “Beyond leadership”: unraveling the impact of ethical leadership on the most influential factors, an analysis of the mediating role of ethical climate and employee moral identity

**DOI:** 10.3389/fpsyg.2026.1870811

**Published:** 2026-07-09

**Authors:** Miluska Villar-Guevara, Elizabeth Emperatriz García-Salirrosas, Ledy Gómez-Bayona, Rosse Canaza-Quispe, Israel Fernández-Mallma

**Affiliations:** 1 Escuela Profesional de Administración, Facultad de Ciencias Empresariales, Universidad Peruana Unión, Juliaca, Peru; 2 Faculty of Management Science, Universidad Autónoma del Perú, Lima, Peru; 3 Instituto Tecnológico Metropolitano, Medellín, Colombia; 4 Escuela Profesional de Educación, Facultad de Ciencias Humanas y Educación, Universidad Peruana Unión, Juliaca, Peru

**Keywords:** ethical leadership, ethical climate, willingness to report ethical problems, affective commitment, workplace happiness, employee moral identity, employee well-being, decent work

## Abstract

**Introduction:**

In the face of increasing global demands and growing concerns regarding organizational sustainability, ethical leadership has emerged as a critical factor shaping employee wellbeing, commitment, and ethical behavior in the workplace. Accordingly, this study aimed to examine the influence of ethical leadership on workplace happiness, affective commitment, willingness to report ethical problems, ethical climate, and employee moral identity, while assessing the mediating roles of ethical climate and employee moral identity. This research is justified by the limited empirical evidence regarding the mechanisms through which ethical leadership influences employee attitudes and behaviors, particularly within integrated explanatory models that simultaneously consider both organizational and individual ethical factors.

**Method:**

The study adopted an explanatory design, with 462 Peruvian workers. Participants were women (64.9%) and men (35.1%), aged between 21 and 64 years (*M* = 36.6; SD = 10.1). The model was statistically analyzed using PLS-SEM.

**Results:**

The hypotheses of the proposed model were confirmed, demonstrating the influence of ethical leadership on workplace happiness, affective commitment, willingness to report ethical problems, ethical climate, and employee moral identity, highlighting in turn the mediating role of the ethical climate and employee moral identity in the proposed model.

**Discussion:**

These findings suggest that ethical leadership functions as an integrative mechanism that links the organizational, psychological, and behavioral dimensions of employee performance. The study contributes to the literature by demonstrating the simultaneous mediating role of ethical climate and employee moral identity in explaining how ethical leadership influences model components. By providing evidence from the Peruvian healthcare sector, a context that is still underrepresented in organizational behavior research, this study expands the current theoretical understanding of ethical leadership. It offers perspectives for strengthening integrity, transparency, and sustainable people management practices in healthcare institutions.

## 1 Introduction

Ethical leadership is considered a topic that has gained greater relevance in the last decade, due to the fact that it has been the subject of study from various contexts, such as the commercial, educational, health, and banking sectors, even impacting non-governmental entities (NGOs) (Hosseini and Ferreira, 2023; Li et al., 2023; Azila-Gbettor et al., 2024). Even though the impact of leadership decisions at the operational, tactical, and strategic levels has been confirmed, wrong decisions have often resulted in loss of institutional prestige, fines and penalties, permanent closures, among other consequences (Strobel et al., 2010; Tutar et al., 2011), this shows the urgency of ethical leadership, since its foundation is moral consistency, presented as a pattern of behavior and decision-making that non-negotiably emphasizes the exercise of principles, ethics, and morality over time (Brown et al., 2005; Brown and Treviño, 2006).

In recent decades, Peru has experienced remarkable economic growth; however, this progress has been accompanied by persistent challenges regarding ethical responsibility and transparency. Simultaneously, the erosion of social principles and values has intensified the demand for organizations seeking leaders with strong ethical foundations, capable of inspiring trust and fostering sustainable organizational growth (Ogaga et al., 2023). In this sense, evidence suggests that leaders with a strong sense of justice and moral identity generate greater trust in their teams, which translates into superior organizational performance and higher levels of self-efficacy among employees (Asif et al., 2019; Castillo et al., 2019).

From a theoretical perspective, ethical leadership has been widely linked to the promotion of positive organizational behaviors. According to social learning theory, leaders act as role models, so a lack of ethics can reinforce individualistic and self-interested behaviors, while ethical conduct promotes cooperation and collective wellbeing (Yukl et al., 2013; Wright et al., 2016). Along these lines, ethical leadership has the capacity to generate positive work environments that strengthen employee motivation, commitment, and loyalty, while also fostering innovation and reducing risk aversion (Hosseini and Ferreira, 2023). In operational terms, administrators, managers, or supervisors who adopt an ethical leadership style provide guidance, resources, and structures that discourage unethical behavior. Likewise, when employees face ethical dilemmas, ethical leaders play a key role by providing competencies and skills that facilitate decision-making aligned with organizational standards, acting as agents of change within the work team (Kuenzi et al., 2020). In this context, ethical leaders are positioned as fundamental actors in fostering an ethical organizational culture, strengthening the ethical climate, and developing employees’ moral identity.

In the healthcare sector, professionals operate in environments characterized by high ethical standards, resource limitations, organizational pressures, and increasing expectations regarding the quality of care. Under such conditions, ethical leadership constitutes a strategic organizational resource capable of strengthening employee wellbeing, promoting affective commitment, and consolidating behaviors aligned with institutional values. Conversely, the absence of adequate management, trained personnel, and ethical practices significantly increases the risk of organizational failure (Meyer-Sahling et al., 2019). In response to these challenges, the literature has documented associations between ethical leadership and variables such as organizational culture, job commitment, moral identity, corporate social responsibility, psychological empowerment, decision-making, and job performance, demonstrating its relevance to the effectiveness and sustainability of organizations.

However, significant knowledge gaps remain regarding the mechanisms that explain how ethical leadership influences employee attitudes and behaviors, especially in Latin American healthcare contexts. Furthermore, the available evidence has paid little attention to the combined effects of critical factors such as ethical climate, employee moral identity, workplace happiness, affective commitment, and willingness to report ethical problems. In this context, the present research contributes to the literature by integrating these variables into a single explanatory framework, simultaneously examining the mediating effects of ethical climate and moral identity. In this way, the study expands the theoretical understanding of ethical leadership. It provides empirical evidence from healthcare workers in Peru, a context still underrepresented in the international literature on organizational behavior and strategic human talent management. In this sense, the objective of the present study was to analyze the influence of ethical leadership on workplace happiness, affective commitment, willingness to report ethical problems, ethical climate, and employee moral identity, examining the mediating role of ethical climate and employee moral identity.

In the following, the present study is divided into the following sections: Section 2 contains the literature review and study hypotheses. Section 3 provides materials and methods. Section 4 focuses on the results. Section 5 refers to the discussion, and Section 6 the conclusions.

## 2 Literature review and study hypotheses

### 2.1 Ethical leadership

The alignment of moral values distinguishes ethical leadership, the display of honesty and fairness, and the creation of an environment where the team feels free to address their dilemmas without worry (Pakizekho and Barkhordari-Sharifabad, 2022). This leadership approach helps to foster ethical actions within teams (Yu et al., 2024). These leaders promote moral awareness by enabling the evaluation of others’ behavior based on self-judgment of their values and norms, inspiring active moral action in ethically complex scenarios (Meng and Guo, 2025). Recent studies show that these leaders help reduce organizational silence, fostering an ethical culture and climate in the workplace (She et al., 2023). Thus, ethical leadership is not only a moral stance, but also becomes a key element in creating work environments where employees feel safe and supported when reporting ethical issues.

### 2.2 Workplace happiness

Workplace happiness refers to a positive psychological state characterized by favorable emotions, cognitions, and motivational dispositions that emerge from employees’ work experiences (Laowanich et al., 2021). Beyond the experience of momentary positive feelings, workplace happiness has been associated with job satisfaction, professional fulfillment, organizational commitment, and employees’ intentions to remain within their organizations. Previous research has identified several organizational factors that contribute to workplace happiness, including compensation systems, communication quality, workplace ergonomics, peer support, supervisory practices, and leadership behaviors, highlighting the central role of leaders in shaping positive work environments (Han, 2023).

Within this framework, ethical leadership has been recognized as a particularly relevant antecedent of workplace happiness because ethical leaders foster trust, fairness, transparency, and respect in interpersonal relationships, thereby creating conditions that promote employee wellbeing and positive workplace experiences. Empirical evidence from healthcare settings indicates that ethical leadership positively influences workplace happiness and related organizational outcomes by strengthening social relationships and enhancing the quality of the work environment (Gonçalves and Curado, 2023). Similarly, recent findings from the Peruvian healthcare sector revealed that employees who perceive their leaders as fair and ethically responsible are more likely to emulate such behaviors, contributing to a more trustworthy organizational climate and higher levels of workplace happiness (Villar-Guevara et al., 2025). Collectively, these findings suggest that ethical leadership constitutes a strategic organizational resource capable of fostering workplace happiness, thereby supporting the hypothesis that ethical leadership positively influences workplace happiness.

#### 2.2.1 Factors related to the job and factors related to the worker

Workplace happiness has been analyzed from various perspectives and theories; however, one of the most applicable approaches is the one that considers two aspects: one based on internal elements such as satisfaction with the tasks performed, and the other based on external components related to working conditions and relationships with others (Del Junco et al., 2013; Ramirez-Garcia et al., 2019; Ramírez-Gañan et al., 2020). These positions have been significantly linked to subjective wellbeing, motivation, job performance, and emotional connection with the company (Sierra et al., 2021). In this sense, happiness in the workplace is described in two components: Factors Related to the Job (FRJ) and Factors Related to the Worker (FRW).

Referring to Factors Related to the Job, these aspects include freedom at work, a sense of duty, and task diversity, all fundamental factors for achieving happiness in the workplace. Freedom at work refers to the degree to which a worker has control over their tasks and work decisions, which is related to increased satisfaction and motivation. A sense of duty, on the other hand, is linked to greater commitment and performance in one’s duties. Task diversity is associated with reduced monotony and increased workplace happiness. Worker-related factors, meanwhile, involve, for example, work motivation and individual capabilities, which are fundamental to employee performance. Scientific literature has shown that intrinsic motivation, related to personal satisfaction, plays a crucial role (Karaferis et al., 2022; Zhou et al., 2023), significantly influencing commitment and effectiveness at work (Schopman et al., 2017; Joe-Akunne et al., 2025; Santiago-Torner et al., 2025a).

### 2.3 Employee moral identity

Employee moral identity is understood as that which determines the extent to which moral traits and values shape a person’s self-concept and guide them towards ethical, morally acceptable conduct that directs their actions in the workplace (Cui et al., 2021; Li et al., 2022). In this sense, employees who possess high indicators of moral identity tend to exhibit fewer negative behaviors and a greater sense of belonging to the organization, which reinforces their commitment to ethics (Kim and Choi, 2021). A recent study demonstrated that ethical leadership potentially influences the ethical behavior of employees when they have a high sense of moral identity and self-discipline (Al Halbusi et al., 2023). On the other hand, workers with a strong moral identity better understand the relationship between corporate social responsibility and workplace safety, resulting in a greater commitment to the company’s standards and values (Hong and Roh, 2024). Researchers in this field of study have made it possible to understand the impact of ethical leadership on the voice of followers and the mediating role that moral identity plays (Zahoor et al., 2025). Furthermore, it has been shown how ethical leadership positively impacts the ethical behavior of employees, and to what extent the effect of ethical leadership on the ethical behavior of employees was greater when leaders displayed a higher moral identity (Alhaidan, 2025).

#### 2.3.1 Moral identity internalization and moral identity symbolization

Moral identity can be analyzed from the perspective of two components: Moral Identity Internalization (MII) and Moral Identity Symbolization (MIS). Internalization plays a fundamental role, where these principles are seen as essential for self-perception, promoting actions consistent with the moral self even without external recognition and functioning as a self-regulation mechanism (Goering et al., 2024). Internalization refers to the extent to which ethical values, such as integrity, fairness, and honesty, are deeply ingrained in how an employee sees themselves. This guides their moral choices without requiring external observation (Li et al., 2022). This shows that moral values serve as a mental foundation for actions that promote the common good. Finally, Ahmed and Khan (2024) found that, in the health field, employees with a strong moral identity responded better to ethical leadership and were less likely to break the rules, putting their moral principles first. On the other hand, the symbolism of these values must be clearly shown, being presented through socially recognized ethical behaviors, testimonial accounts, or the use of corporate emblems in the workplace (Kim and Choi, 2021). This facet implies that people express their convictions and principles in social situations to strengthen their moral identity and receive recognition from others. On the other hand, Xu et al. (2024) emphasize that symbolization is a distinctive aspect of moral identity that focuses on transmitting values to others, unlike internalization, which involves a private and personal assimilation of those values.

### 2.4 Ethical climate

Ethical climate is defined as the set of shared perceptions within an organization about what is considered morally right or wrong and how ethical dilemmas that arise in the workplace should be addressed (Rambod et al., 2024). The ethical climate is seen as the set of rules, values, and shared understandings that guide appropriate behavior within a workplace. This concept influences how people address moral issues in their work environment, serving as a moral compass (Tannorella et al., 2022). On the other hand, developing positive ethical climates reduces organizational cynicism, provided there is a strong link between the values of the individual and those of the organization, which in turn strengthens organizational pride and affective commitment (Wnuk, 2025). This is not limited to written rules but reflects a lived ethical culture that guides the daily behavior of staff. Fostering a positive ethical climate strengthens critical thinking and improves job performance by providing an environment where ethical decisions are made with clarity and institutional support (Park and Jeong, 2024). In that sense, the strong link between leadership and ethical climate has been demonstrated (Chen and Hou, 2016; Freire and Pinto, 2022; Gwamanda and Mahembe, 2023; Yang et al., 2023; Azila-Gbettor et al., 2024). In this regard, recent studies have clarified and validated the impact of ethical leadership on the design of ethical climates in work environments and have suggested a management and corporate culture model that benefits both the company and those who are part of it (Özden et al., 2019; Al Halbusi et al., 2021; Zhang et al., 2026).

### 2.5 Affective commitment

Affective commitment is a form of positive emotional bond that an employee develops toward their organization, reflecting identification, involvement, and a voluntary desire to remain. This type of commitment is strengthened in organizational contexts where perceived support is high, especially through recognition, equity, and open communication (Shan et al., 2023). Affective commitment is recognized as the emotional bond that an employee feels towards their employer, creating a strong connection based on trust and loyalty (Paparisabet et al., 2024). From the perspective of Social Exchange Theory, it is said that if a company supports your growth and ideas, then you feel you should give something in return, and this makes you like your job more (Medina-Garrido et al., 2023). In contrast, studies show that those who are highly emotionally invested in their work suffer less from emotional exhaustion and tolerate higher levels of work-related stress (Stark et al., 2025). Therefore, an ethical climate helps strengthen this emotional connection by fostering a fair, empathetic organizational environment focused on collective wellbeing. Consequently, affective commitment has become an emotional driver strongly linked to ethical leadership (Santiago-Torner et al., 2024, 2025b; Tran, 2024); the latter has both a direct and indirect effect on affective commitment and the intention to rotate (Demirtas and Akdogan, 2015; Ngang and Raja, 2015). Furthermore, affective commitment has also been validated as acting as a mediating mechanism (Santiago-Torner et al., 2024) in the relationship between ethical leadership and work commitment (Asif et al., 2019), and with civic behavior (Anwar et al., 2012).

### 2.6 Willingness to report ethical problems

The willingness to report ethical problems refers to employees’ desire to alert their supervisor, manager, or senior management about inappropriate conduct in their work environment, a key factor in maintaining an effective ethical culture. In this regard, a study demonstrated that ethical leadership, by modeling transparency and fairness, reduces fear of retaliation and promotes this type of reporting (Nguyen et al., 2025). In this sense, employees perceive an organizational culture that is perceived as ethical, their professional commitment is strengthened, and their willingness to report irregularities is increased (Abbasi and Amiri, 2025). Furthermore, ethical leadership increases the moral empowerment of employees, which in turn strengthens their willingness to report ethical problems, especially when there is psychological safety in the work environment (Sumanth et al., 2024).

In contrast, employees operating within hierarchical organizational cultures often exhibit lower levels of trust in internal mechanisms designed to report ethical concerns and safeguard confidentiality (Świątek-Barylska and Braila, 2024). Conversely, evidence suggests that confidential reporting systems reduce fears of retaliation while enhancing employees’ perceptions of organizational justice (Smaili, 2023). Moreover, the development of a strong ethical climate fosters an organizational environment that strengthens employees’ identification with the organization, which, in turn, significantly increases their willingness to report unethical or inappropriate conduct (Cai et al., 2024). Taken together, these studies indicate that the willingness to report ethical problems depends as much on organizational structure and available resources as on the cultural context, all of which are essential to fostering transparency and institutional integrity.

Based on the above, the following hypothesis is proposed:

*H1*: Ethical leadership influences workplace happiness.

*H2*: Ethical leadership influences affective commitment.

*H3*: Ethical leadership influences willingness to report ethical problems.

*H4*: Ethical leadership influences ethical climate.

*H5*: Ethical leadership influences employee moral identity.

*H6*: Ethical climate mediates the relationship between ethical leadership and affective commitment.

*H7*: Ethical climate mediates the relationship between ethical leadership and workplace happiness.

*H8*: Employee moral identity mediates the relationship between ethical leadership and willingness to report ethical problems.

**Figure 1:**
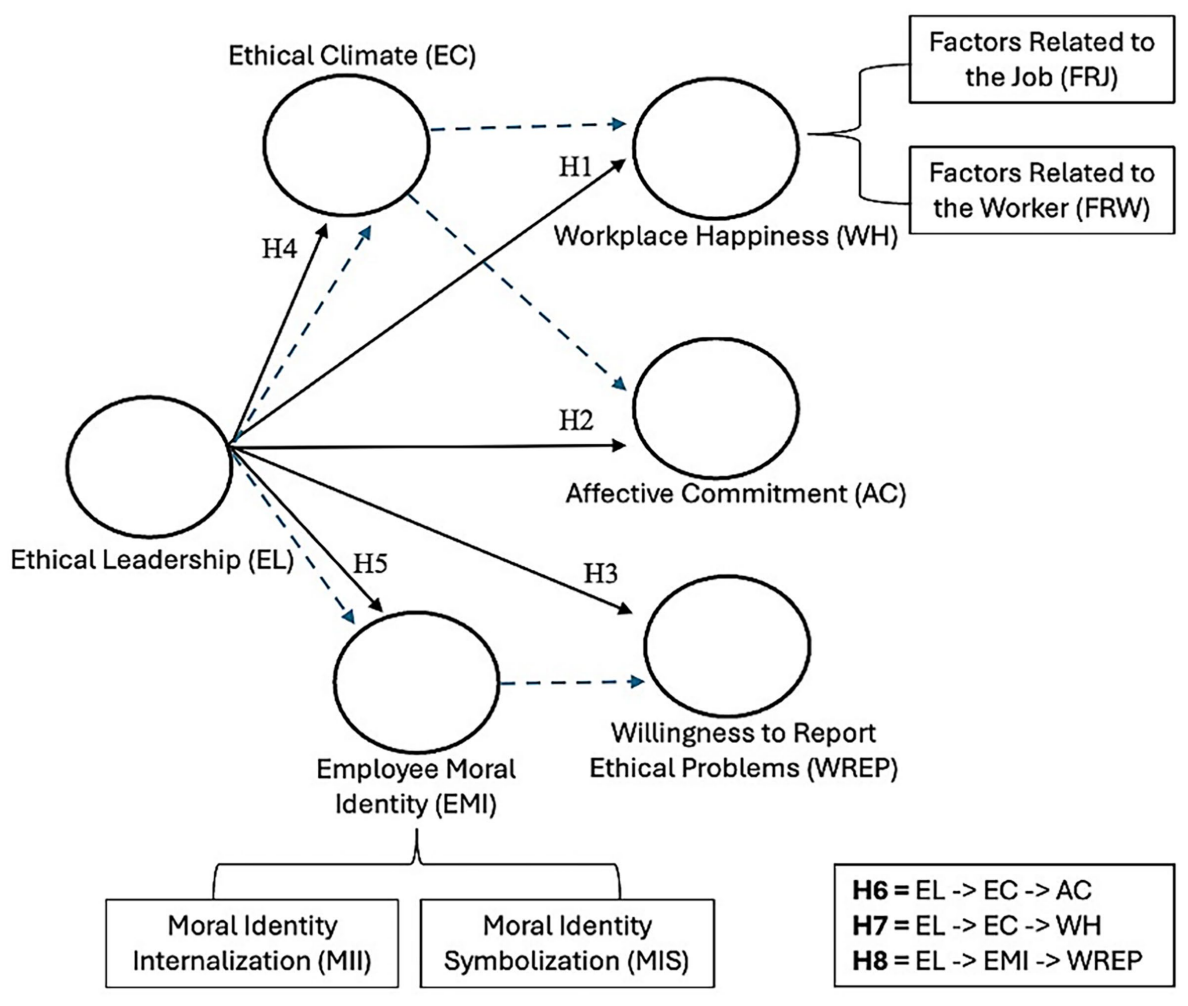
Proposed hypothetical model.

Taking into account the hypotheses mentioned above, the conceptual model resulting from the study can be visualized in the graphic representation of Figure 1.

## 3 Materials and methods

### 3.1 Study design

It was considered an explanatory study (Ato et al., 2013).

### 3.2 Participants

The study population consisted of Peruvian healthcare workers. Only individuals who met the inclusion criteria established from the initial research idea were considered. These criteria included being of legal age (minimum 18 years), working in the administrative and/or healthcare area of a public or private entity in Peru, and having been employed at that institution for a minimum of 6 months at the time of completing the questionnaire. Those who did not meet these inclusion criteria were excluded. The surveys were self-administered by each participant. Using non-probability convenience sampling, 462 workers (90% response rate) between 21 and 64 years of age (*M* = 36.61, SD = 10.15), including both sexes, participated voluntarily. These participants were contacted by visiting representative public and private healthcare facilities in Peru. Institutional permission was granted to staff, who were granted free access during weekly report meetings and month-end closing meetings. In this regard, the online questionnaire was shared with them via QR code and/or direct WhatsApp links. Table 1 shows that the majority of participants were women (64.9%), single (31.0%), between 21 and 39 years old (63.9%), and had worked for a health entity for no more than 5 years (64.9%).

**Table 1:** Sociodemographic profile of 462 participants.

Characteristics	Category	Frequency	Percentage %
Sex	Female	300	64.9
	Male	162	35.1
Marital status	Single	143	31.0
	Cohabitant	137	29.7
	Married	132	28.6
	Divorced	27	5.8
	Widow(er)	23	5.0
Age range	21–39 years	295	63.9
	40–64 years	167	36.1
Time spent working for the institution	Up to 5 years	300	64.9
	6 to 10 years	97	21.0
	11 years and older	65	14.1

### 3.3 Back-translation and validation process

All scales, except those used to measure Ethical Leadership (EL) and Workplace Happiness (WH), were adapted from their original language (English) to Spanish and contextualized for Peruvian healthcare personnel through a back-translation process. Subsequently, semantic validation was performed through an online focus group session, allowing for semantic modifications to the work context and the actual study sample. Six workers who met the inclusion criteria and other experts in the field of study participated in this session. Each participant provided feedback on the wording of each item, leading to semantic adjustments until the questionnaire was ready for application. Additionally, to assess the quality of the reflective constructs (Table 2), convergent validity and reliability, that is, the internal consistency of all constructs, were analyzed. These procedures were considered sufficient for the objective of this study.

**Table 2:** Validation of the first and second order measurement model with reliability and convergent validity.

Construct	Code	Outer loadings	*α*	Composite reliability (rho_c)	AVE
First order					
Affective Commitment (AC)	AC1	0.837	0.850	0.899	0.689
	AC2	0.825			
	AC3	0.829			
	AC4	0.830			
Ethical Climate (EC)	EC1	0.846	0.881	0.913	0.678
	EC2	0.806			
	EC3	0.837			
	EC4	0.800			
	EC5	0.826			
Ethical Leadership (EL)	EL1	0.827	0.944	0.952	0.689
	EL2	0.815			
	EL3	0.867			
	EL4	0.823			
	EL5	0.827			
	EL6	0.821			
	EL7	0.822			
	EL8	0.823			
	EL9	0.846			
Factors Related to the Job (FRJ)	FRJ1	0.865	0.931	0.944	0.706
	FRJ2	0.812			
	FRJ3	0.847			
	FRJ4	0.845			
	FRJ5	0.840			
	FRJ6	0.837			
	FRJ7	0.836			
Factors Related to the Worker (FRW)	FRW1	0.842	0.853	0.901	0.695
	FRW2	0.841			
	FRW3	0.786			
	FRW4	0.863			
Moral Identity Internalization (MII)	MII1	0.829	0.797	0.850	0.539
	MII2	0.770			
	MII3	0.864			
	MII4	0.563			
	MII5	0.593			
Moral Identity Symbolization (MIS)	MIS1	0.828	0.866	0.903	0.651
	MIS2	0.779			
	MIS3	0.856			
	MIS4	0.760			
	MIS5	0.808			
Willingness to Report Ethical Problems (WREP)	WREP1	1.000			
Second order					
Workplace Happiness (WH)	FRJ	0.906	0.750	0.889	0.800
	FRW	0.882			
Employee Moral Identity (EMI)	MII	0.871	0.716	0.875	0.778
	MIS	0.893			

### 3.4 Data collection instruments

For this process, a virtual questionnaire was designed and hosted on the Google Forms platform. This questionnaire was divided into three parts. The first section included instructions for completing the questionnaire along with a brief consent form. The second section requested sociodemographic information from the participants, and finally, the measurement scales were presented (Appendix A). The questionnaire used a 5-point Likert scale for all items, ranging from 1 (Strongly Disagree) to 5 (Strongly Agree). First, to measure ethical leadership, the Ethical Leadership Questionnaire (ELQ) was used, a one-dimensional scale of only 9 items (*α* = 0.944), taken from a study applied in Peru (Villar-Guevara et al., 2025). Secondly, to measure Workplace Happiness, a metric applied to the Peruvian population was used (Agustin-Silvestre et al., 2024; Villar-Guevara et al., 2025), consisting of 11 items with a two-dimensional structure (*α* = 0.931 and 0.853, respectively): Factors Related to the Job (FRJ) and Factors Related to the Worker (FRW). Furthermore, the metric used by Al Halbusi et al. (2023) was employed to measure Employee Moral Identity, which consists of 10 items with a 2-dimensional structure (*α* = 0.797 and 0.866, respectively): Moral Identity Internalization (MII) and Moral Identity Symbolization (MIS). The study by Yang et al. (2023) was used to measure Ethical Climate and consisted of 5 items with a one-dimensional structure (*α* = 0.881). Subsequently, to measure Affective Commitment, a 4-item scale with a unidimensional structure was used (*α* = 0.850). Finally, Willingness to Report Ethical Problems was measured in a single item (*α* = 1.00). These last metrics were taken from a previous study (Wright et al., 2016).

### 3.5 Ethical considerations

This study complied with the standards established by the academic unit of a private university in Peru. Prior to its implementation, it was reviewed, improved, and approved by the Ethics Committee of the Graduate School of the Universidad Peruana Unión (approval code 2024-CEEPG-00020, dated February 16, 2024). Before data collection, participants were instructed on how to complete the online questionnaire, the purpose of the study, and informed about their voluntary and anonymous participation and the confidentiality of their information. Throughout the study process, care was taken to ensure compliance with the 1964 Declaration of Helsinki. Informed consent was obtained from each participant through the following declaration: “I acknowledge that by completing this questionnaire, I am giving my consent to participate in the study.” The participation link was available from February 27, 2024, for approximately 6 months.

### 3.6 Statistical analysis

The study conducted a two-stage statistical analysis: first, the measurement model was evaluated, and then the structural model. As part of the initial process, IBM SPSS version 25 was used to examine the sociodemographic data of the study participants, which are shown in Table 1. For the statistical analysis of the data, the partial least squares structural equation modeling (PLS-SEM) method was employed using SmartPLS version 4.0 software. This method was selected due to its robustness when working with complex models, moderate sample sizes, and formative or reflective latent variables.

The evaluation process of the measurement model examined three indicators: (1) internal consistency using Cronbach’s alpha (*α*) and composite reliability (CR); (2) convergent validity and mean variance extracted (AVE) of the constructs; and (3) discriminant validity of the constructs, using the Fornell-Larcker scale and the Heterotrait-Monotrait (HTMT) criteria. Finally, a structural model analysis was performed to evaluate the proposed hypotheses. First, it was determined whether the relationships defined in the model were significant; for this, the *p*-value had to be less than 0.05. This approach allowed for the validation of the proposed model and a comprehensive analysis of the relationships between the latent variables (Hair et al., 2019).

To test for the potential presence of Common Method Variance (CMV), Harman’s single-factor test was performed. The 44 items from this study were included in an exploratory factor analysis using principal component extraction without rotation. The first factor without rotation in the exploratory factor analysis explained 41.67% of the total variance, which is below the 50% cutoff point recommended by Podsakoff et al. (2003). This leads us to conclude that CMV is unlikely to introduce bias into this study. Furthermore, as a procedural measure, participant anonymity was guaranteed, participation was voluntary, and the study’s objective was clearly communicated before data collection. This approach aimed to reduce the possibility of social desirability bias (Podsakoff et al., 2003).

## 4 Results

### 4.1 Validation of the measurement model

The validity and reliability analysis of the measurement model revealed satisfactory psychometric indicators that support the robustness of the constructs assessed. Composite reliability ranged from 0.853 to 0.931, significantly exceeding the recommended threshold of 0.70 (Hair et al., 2019); this demonstrates the internal consistency of the scales used. Furthermore, Cronbach’s alpha (α) values ranged from 0.789 to 0.902, confirming the reliability of the measurement instruments. Regarding convergent validity, the Average Variance Extracted (AVE) for all constructs exceeded the minimum criterion of 0.50 (Fornell and Larcker, 1981), with values ranging between 0.536 and 0.706, which indicates that each construct explains more than half of the variance of its respective indicators.

The second-order constructs showed factor loadings greater than 0.70 in their component dimensions, meeting the established standards for reflective models (Chin, 1998). In particular, Ethical Leadership (EL) showed composite reliability values of 0.952 and an AVE of 0.689, confirming its validity as a unidimensional construct. These results are consistent with previous research that has validated ethical leadership scales in Latin American organizational contexts (Brown et al., 2005; Kalshoven et al., 2011) and provide empirical evidence of the model’s applicability in the Peruvian healthcare sector (See Table 2).

### 4.2 Discriminant validity

#### 4.2.1 First-order discriminant validity

The discriminant validity of the model was assessed using the Heterotrait-Monotrait Ratio (HTMT) criterion, the results of which confirmed that the first-order constructs are empirically distinct from one another. All HTMT values were below the conservative threshold of 0.85 (Henseler et al., 2015); the maximum value of 0.708 was found between Moral Identity Symbolization (MIS) and Affective Commitment (AC), indicating no collinearity issues among the latent variables. The lowest values were observed between Willingness to Report Ethical Problems (WREP) and Moral Identity Symbolization (MIS) (0.218), as well as between WREP and Moral Identity Internalization (MII) (0.218), suggesting a clear conceptual distinction between economic perceptions and intrinsic motivational dimensions. These findings are consistent with the postulates of Henseler et al. (2015) and Gold et al. (2001), who emphasize that HTMT values below 0.90 provide robust evidence that each construct captures unique and non-redundant phenomena, thus validating the specification of the measurement model in the context of Peruvian health workers (see Table 3).

**Table 3:** Discriminant validity matrix of the first-order HTMT relationship.

Construct	AC	EC	EL	FRJ	FRW	MII	MIS	WREP
AC								
EC	0.680							
EL	0.626	0.596						
FRJ	0.595	0.684	0.632					
FRW	0.658	0.659	0.567	0.673				
MII	0.533	0.678	0.535	0.575	0.530			
MIS	0.708	0.647	0.580	0.550	0.626	0.641		
WREP	0.299	0.329	0.378	0.368	0.325	0.218	0.318	

#### 4.2.2 Second-order discriminant validity

According to Table 4, the discriminant validity analysis for the second-order constructs using the HTMT criterion revealed that, although the variables exhibit conceptual differentiation, there are considerable correlations between some key constructs of the model. The highest values were observed between Employee Moral Identity (EMI) and Workplace Happiness (WH) at 0.892, followed by Employee Moral Identity (EMI) and Ethical Climate (EC) at 0.833, and Workplace Happiness (WH) with Ethical Climate (EC) reaching 0.820, all of them approaching the critical threshold of 0.90 established by Henseler et al. (2015). Although these values do not exceed the permissible limit, they suggest a close theoretical relationship between Ethical Climate (EC), Employee Moral Identity (EMI), and Workplace Happiness (WH) in healthcare workers, consistent with the literature that postulates that these constructs are part of an interconnected system of personal resources and positive psychological states (Ryan and Deci, 2000; Schaufeli and Bakker, 2004). On the other hand, Willingness to Report Ethical Problems (WREP) showed the lowest HTMT values with all constructs (range: 0.299–0.423), confirming its conceptual independence and discriminant validity with respect to the psychological variables of the model, which supports the theoretical specification that economic perceptions constitute a distinct domain of subjective wellbeing in work contexts (Diener and Biswas-Diener, 2002).

**Table 4:** Discriminant validity matrix of the second-order HTMT relationship.

Construct	AC	EC	EL	EMI	WH	WREP
AC						
EC	0.680					
EL	0.626	0.596				
EMI	0.794	0.833	0.714			
WH	0.764	0.820	0.733	0.892		
WREP	0.299	0.329	0.378	0.355	0.423	

### 4.3 Evaluation of the structural model

**Figure 2:**
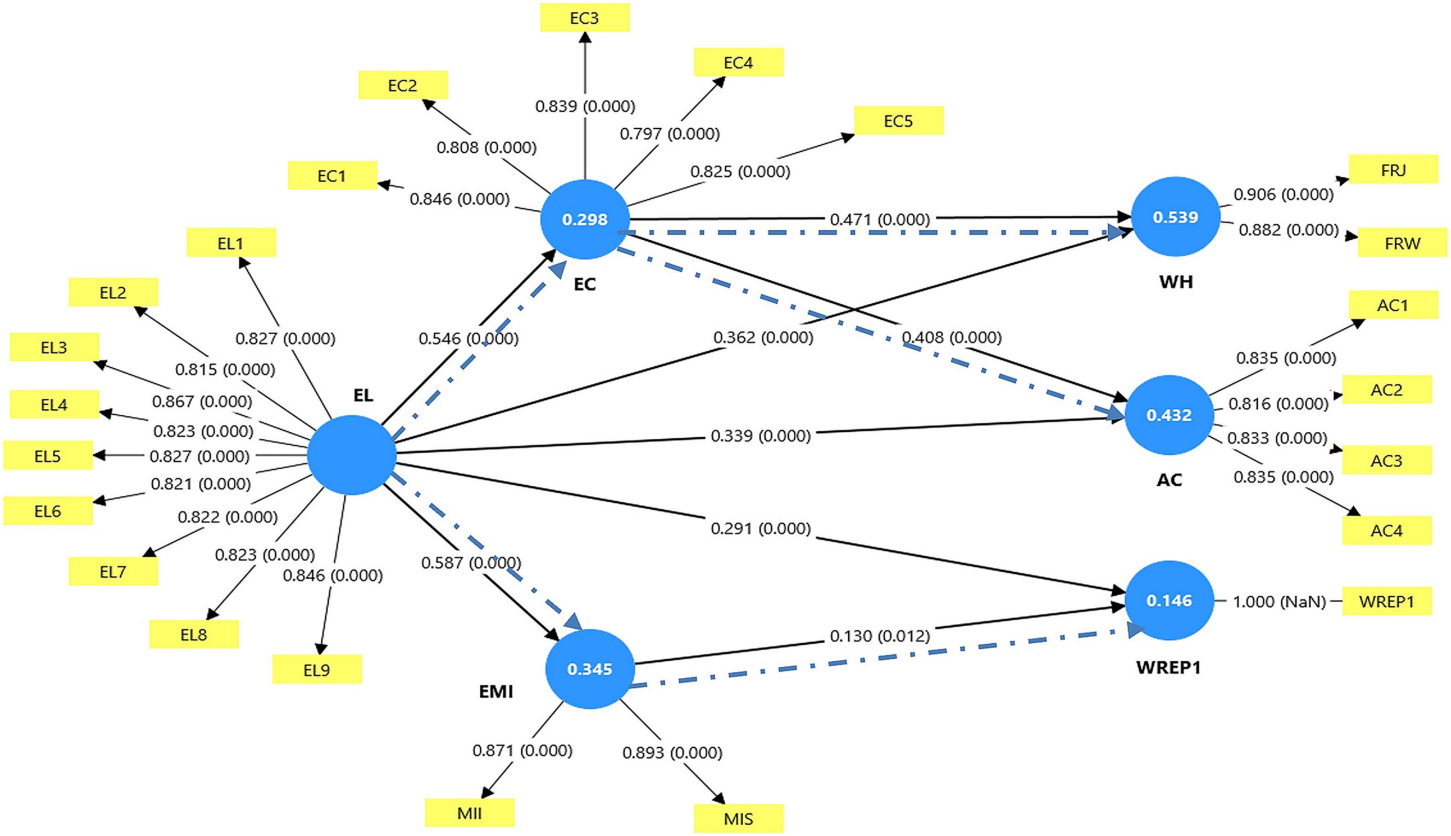
Structural model. Note. Solid black arrows indicate direct effects; blue dashed arrows indicate mediation paths.

The structural model (Figure 2) demonstrates the causal relationships between Ethical Leadership (EL) and the resulting organizational wellbeing variables in Peruvian healthcare workers. The results reveal that Ethical Leadership (EL) has a direct, significant, and substantial effect on Ethical Climate (EC) (*β* = 0.546, *p* < 0.001) and on Employee Moral Identity (EMI) (*β* = 0.587, *p* < 0.001), confirming the postulates of social learning theory, which maintains that ethical leaders model behaviors that foster psychological activation and self-determined motivation in their followers (Brown and Treviño, 2006; Kalshoven et al., 2011). Likewise, significant indirect effects of Ethical Leadership (EL) on Workplace Happiness (WH) (*β* = 0.471, *p* < 0.001) and Affective Commitment (AC) (*β* = 0.339, *p* < 0.001) were observed, mediated mainly by engagement, which is consistent with the Job Demands and Resources (JD-R) model that proposes that social resources, such as Ethical Leadership (EL), generate positive motivational states that in turn promote wellbeing outcomes (Bakker and Demerouti, 2017). Notably, the relationship between Employee Moral Identity (EMI) and Willingness to Report Ethical Problems (WREP) was not significant (*β* = 0.130, *p* = 0.912), suggesting that intrinsic motivation and economic perceptions operate as independent dimensions of subjective wellbeing, supporting the theoretical distinction between hedonic and eudaimonic wellbeing proposed by Ryan and Deci (2001).

### 4.4 Hypothesis testing

The results of the hypothesis testing (Table 5) confirm the acceptance of all the relationships proposed in the theoretical model, highlighting the central role of Ethical Leadership (EL) as a predictor of positive organizational outcomes in the Peruvian health context. The hypotheses postulating direct effects of Ethical Leadership (EL) showed highly significant t-values: EL → EMI (*t* = 15.746, *p* < 0.001, *β* = 0.587), EL → EC (*t* = 12.021, *p* < 0.001, *β* = 0.546), EL → AC (*t* = 6.398, *p* < 0.001, *β* = 0.339), EL → WH (*t* = 7.378, *p* < 0.001, *β* = 0.362), and EL → WREP (*t* = 4.770, *p* < 0.001, *β* = 0.291), confirming that Ethical Leadership (EL) constitutes a fundamental social resource that drives both motivational states and objective dimensions. Subjective aspects of wellbeing, in line with the postulates of the conservation of resources theory (Hobfoll, 2001) and the social exchange theory (Blau, 1964). Additionally, the proposed mediation effects were corroborated: EC → AC (*t* = 6.227, *p* < 0.001, *β* = 0.223), EC → WH (*t* = 8.044, *p* < 0.001, *β* = 0.257), and EMI → WREP (*t* = 2.390, *p* = 0.017, *β* = 0.077), which suggests that the Ethical Climate (EC) and intrinsic motivation operate as psychological mechanisms through which Ethical Leadership (EL) translates into tangible wellbeing outcomes, consistent with the JD-R model that postulates that work resources generate chain motivational processes (Schaufeli and Bakker, 2004; Bakker and Demerouti, 2017).

**Table 5:** Hypothesis testing.

H	Hypothesis	Original sample (O)	Sample mean (M)	Standard deviation (STDEV)	*T* statistics (|O/STDEV|)	*p* values	Decision
H1	EL - > WH	0.362	0.362	0.049	7.378	0.000	Accepted
H2	EL - > AC	0.339	0.339	0.053	6.398	0.000	Accepted
H3	EL - > WREP	0.291	0.288	0.061	4.770	0.000	Accepted
H4	EL - > EC	0.546	0.546	0.045	12.021	0.000	Accepted
H5	EL - > EMI	0.587	0.589	0.037	15.746	0.000	Accepted
H6	EL - > EC - > AC	0.223	0.224	0.036	6.227	0.000	Accepted
H7	EL - > EC - > WH	0.257	0.256	0.032	8.044	0.000	Accepted
H8	EL - > EMI - > WREP	0.077	0.078	0.032	2.390	0.017	Accepted

## 5 Discussion

### 5.1 Discussion of the results

This study aimed to analyze the influence of ethical leadership on workplace happiness, affective commitment, willingness to report ethical problems, ethical climate, and employee moral identity, examining the mediating role of ethical climate and employee moral identity. The results confirm the relevance of ethical leadership as a key organizational factor, capable of generating positive effects on both individual psychological variables and ethical behaviors within the healthcare environment.

The findings of this study have demonstrated that ethical leadership exerts a significant influence on workplace happiness and employee engagement. This finding is consistent with previous research, such as that of Pakizekho and Barkhordari-Sharifabad (2022), who indicate that leaders who act with integrity, fairness, and transparency contribute to creating work environments characterized by trust and psychological safety. These conditions promote employee wellbeing and strengthen their emotional connection to the organization (Meng and Guo, 2025). From the perspective of the labor demands-resources model, ethical leadership can be understood as an organizational resource that facilitates positive motivational states and promotes wellbeing at work (Bakker and Demerouti, 2017). This aspect is particularly relevant in the healthcare sector, where high job demands, emotional pressure, and the responsibility associated with patient care often generate high levels of stress. In this context, the presence of leaders who act ethically can contribute to reducing organizational tensions and promoting healthier and more meaningful work environments.

Similarly, the study results show that ethical leadership significantly influences employees’ willingness to report ethical problems. This is supported by the findings of Nguyen et al. (2025) and Sumanth et al. (2024), who argue that ethical leaders act as role models within organizations, reducing fear of retaliation and fostering responsible reporting. In the healthcare sector, this finding is particularly relevant, as open communication about irregularities is fundamental to ensuring patient safety and improving the quality of care. In this sense, ethical leadership not only fulfills a moral regulatory function within the organization but also promotes transparency and strengthens institutional mechanisms aimed at preventing inappropriate conduct.

The findings of this study allow a more precise understanding of how ethical leadership operates within healthcare organizations. Specifically, the results indicate that ethical leadership does not only exert a direct influence on workplace happiness, affective commitment, and willingness to report ethical problems, but also activates organizational and psychological mechanisms that explain these relationships. The strong effect of ethical leadership on ethical climate (*β* = 0.546) suggests that leaders shape employees’ collective perceptions regarding fairness, transparency, and acceptable conduct within the organization, which is consistent with previous studies emphasizing that ethical climates emerge through leaders’ behaviors and moral modeling (Chen and Hou, 2016; Freire and Pinto, 2022; Yang et al., 2023). In practical terms, this implies that healthcare workers are more likely to experience trust and organizational support when leaders consistently demonstrate ethical conduct.

Likewise, the significant relationship between ethical leadership and employee moral identity (*β* = 0.587) provides evidence that ethical leaders reinforce employees’ internalization of moral values through social learning processes (Brown and Treviño, 2006; Al Halbusi et al., 2023). This result helps explain why employees become more willing to report ethical problems when ethical leadership strengthens moral self-regulation mechanisms. In healthcare settings, where professionals frequently face ethical dilemmas and situations involving patient safety, this process becomes especially relevant because moral identity may function as an internal ethical compass guiding professional conduct.

Furthermore, the mediating role of ethical climate in the relationship between ethical leadership, workplace happiness, and affective commitment demonstrates that employee wellbeing is not explained exclusively by leader behavior itself, but also by the organizational environment that leaders help create. This finding supports the Job Demands–Resources model (Bakker and Demerouti, 2017), which proposes that organizational resources generate motivational states and positive work outcomes. Thus, ethical leadership contributes indirectly to employee wellbeing by fostering a climate characterized by trust, justice, and psychological safety. According to these two hypotheses regarding the mediating role of ethical climate, the results align with the scientific literature, which suggests that collective perceptions of fairness, organizational support, and shared responsibility significantly influence the psychological wellbeing of workers (Park and Jeong, 2024; Wnuk, 2025). Consequently, ethical leadership not only directly impacts employees but also transforms the organizational context in which they work, creating conditions that promote the design of happy and healthy work environments and strengthen identification with the organization.

While the mediating role of employee moral identity in the relationship between ethical leadership and willingness to report ethical problems was confirmed, its effect was marginal, requiring caution in its practical interpretation. This suggests that the internalization of ethical values alone is insufficient to activate reporting behavior, which depends more heavily on organizational factors such as the existence of secure reporting channels, institutional trust, and the perception of protection from retaliation (Smaili, 2023; Świątek-Barylska and Braila, 2024). Future research should examine moderating variables, such as psychological safety and organizational justice, which could amplify the role of moral identity in this behavior (Near and Miceli, 1985; Mesmer-Magnus and Viswesvaran, 2005). Although the mediating effect of employee moral identity on willingness to report ethical problems was statistically significant (*β* = 0.077, *p* = 0.017), the magnitude of the effect was comparatively weaker than the effects observed for ethical climate. This suggests that employees’ willingness to report ethical problems depends not only on internal moral values, but also on contextual organizational conditions such as psychological safety, institutional trust, and protection against retaliation.

This study suggests that ethical leadership operates as an integrating mechanism that connects organizational, psychological, and behavioral dimensions within the health sector. In particular, the findings indicate that fostering ethical leadership styles can be an effective strategy to strengthen the institutional ethical culture, improve workplace wellbeing, and promote responsible behaviors among health professionals. Incorporating ethical leadership as a strategic component in the management of health institutions is especially relevant, particularly in contexts where organizational trust and patient safety depend largely on the integrity and transparency of the professionals who make up the health system.

### 5.2 Theoretical and managerial implications of the study

This study offers theoretical implications that contribute to the literature on ethical leadership, as well as on workplace wellbeing and ethical organizational behavior. From a theoretical perspective, the findings strengthen and deepen the understanding of ethical leadership, showing that it not only acts as a means to prevent misconduct, but also as an organizational resource that enables the activation of affective, relational, and ethical outcomes in the healthcare environment. Analyzing the behavior of the variables in this study reveals two mechanisms through which ethical leadership translates into organizational results. The first is a contextual route, expressed in the ethical climate of the organization; and the second is an individual route, manifested by the moral identity of the employee/collaborator. We highlight this difference because it allows us to understand the effects of ethical leadership, which depend not only on the leader’s behavior, but also on how that behavior shapes the team’s perceptions and activates processes of moral self-regulation.

Following the same line of theoretical contribution, this study links two fields that are generally studied independently: organizational ethics and workplace wellbeing. The findings suggest that ethically led work environments, in addition to promoting the prevention of inappropriate conduct, also strengthen positive work experiences such as workplace happiness and affective commitment. These theoretical contributions are significant because they offer empirical evidence from a Latin American and Peruvian perspective, expanding discussions on ethical leadership in healthcare contexts, where institutional trust, transparency, and patient safety depend largely on the ethical conduct of leaders and staff.

On the other hand, regarding practical implications, the results of this study suggest that healthcare institutions should conduct periodic assessments of the perception of fairness, transparency, and ethical consistency within their work teams. This could be done through internal diagnostics, specific action protocols, and by creating spaces for discussing various work-related and patient care dilemmas. Similarly, healthcare institutions could offer confidential and secure channels for reporting ethical issues, protecting workers from retaliation and institutional repercussions. The presence of ethical leaders can strengthen the willingness to report, but this will only be possible if workers perceive that reporting irregularities will not affect their job security or reputation.

### 5.3 Limitations and future research

Although this research makes an important contribution to business management, some limitations are recognized that should be considered later. First, the cross-sectional design of this study has inherent limitations. Because the data were collected at a single point in time, the study does not allow for examining the temporal evolution or directional dynamics of ethical leadership, ethical climate, willingness to report ethical problems, affective commitment, workplace happiness, and employee moral identity. These constructs can fluctuate over time in response to changes in psychosocial, work, and leadership conditions. Therefore, future research could employ longitudinal designs to investigate the evolution of the model’s behavior, providing a more robust understanding of its stability, interaction patterns, and potential causal mechanisms.

Second, although non-probability convenience sampling is commonly used in this type of study, it introduces a potential self-selection bias that can influence the variety of responses. Therefore, it is suggested that this research be repeated in the future using probability sampling to minimize the risk of bias. Similarly, it is proposed that the analysis be complemented with qualitative methods to explore in depth the mechanisms behind the observed phenomenon and to understand, in a more contextual way, the perceptions, experiences, and meanings that the participants attribute to it. Combining mixed methods approaches would facilitate a more comprehensive and robust understanding of the construct under examination, enriching the interpretation of the empirical results.

Third, the scale used to measure willingness to report ethical problems (WREP) was assessed using a single item. While single-item measures may be appropriate for capturing specific, concrete, and conceptually limited constructs, they inherently restrict the assessment of internal consistency reliability. They may not fully capture the multidimensional nuances underlying intentions to report. Consequently, the observed relationships should be interpreted with caution, as some degree of measurement error may remain unexplained. It is recommended that future research operationalize this construct using multi-item scales that allow for a more complete assessment of its conceptual domain and provide stronger evidence of reliability, convergent validity, and discriminant validity within structural models. Furthermore, it is suggested that future studies include additional variables such as psychological safety, organizational justice, institutional trust, and fear of retaliation, as well as the use of cross-sectoral studies and cross-cultural comparisons.

Finally, because the data were collected from the same participants via a self-report questionnaire, common method variance (CMV) could be a concern. To address this, Harman’s one-factor test was performed; the first unrotated factor explained 41.67% of the total variance, falling below the 50% threshold (Podsakoff et al., 2003), meaning that CMV does not affect the validity of the results. However, future studies should consider multi-informant or longitudinal designs to mitigate this limitation further.

## 6 Conclusion

Leadership has always played a vital role in the workplace, contributing to a more competitive and effective business world. This same principle must be incorporated across various sectors, including healthcare institutions. Consequently, given the context of organizational behavior and increasing global demands, ethical leadership has become a key factor in workplace happiness, engagement, climate, identity, and sustainability. Despite growing evidence of its individual benefits, research on its comprehensive effects on employee wellbeing and ethical behavior, particularly in diverse organizational contexts, is limited. This gap underscores the need to explore the underlying mechanisms by which ethical leadership shapes key employee outcomes. In this regard, this research focused on analyzing the influence of ethical leadership on workplace happiness, affective commitment, willingness to report ethical problems, ethical climate, and employee moral identity, examining the mediating role of ethical climate and employee moral identity. In response, the hypotheses of the proposed model were confirmed.

Finally, this study suggests that ethical leadership operates as an integrative mechanism connecting organizational, psychological, and behavioral dimensions. Incorporating this leadership approach as a strategic component in the management of Peruvian healthcare institutions is especially relevant for professional integrity and transparency. Furthermore, the study provides valuable insights for leaders of healthcare entities interested in fostering ethical leadership among their employees, preventing inappropriate and illegal conduct, as well as sanctions and damage to the institution’s reputation, its members, or its stakeholders.

## Data Availability

The original contributions presented in the study are included in the article/Supplementary material, further inquiries can be directed to the corresponding author.
