# Supplementary material for: “Beyond leadership”: unraveling the impact of ethical leadership on the most influential factors, an analysis of the mediating role of ethical climate and employee moral identity
Source: Front Psychol. 2026 Jul 9;17:1870811. doi: 10.3389/fpsyg.2026.1870811 (PMC13391295; doi:10.3389/fpsyg.2026.1870811)
Supplement: Supplementary file 1 [file Data_Sheet_1.docx]

**Appendix A.** Scales used in Spanish and English

**Liderazgo Ético - Ethical Leadership (EL)**

Mi supervisor/jefe inmediato... *[My immediate supervisor/manager...]*

EL1_Es coherente con sus valores entre lo que dice y lo que hace. *[Maintains its actions consistent with its stated values]*

EL2_Muestra mucha preocupación por los valores éticos y morales. *[Shows great concern for ethical and moral values]*

EL3_Da ejemplo de comportamiento ético en sus decisiones y acciones. *[Sets an example of ethical behavior in your decisions and actions]*

EL4_Es honesto y se puede confiar en que siempre dirá la verdad. *[He is honest and can be trusted always to tell the truth]*

EL5_Fomenta que sus trabajadores adopten prácticas éticas en el trabajo. *[Helps workers to use ethical practices in their work]*

EL6_Insiste en hacer lo justo y ético, incluso cuando no es fácil. *[Insists on doing what is right and ethical even when it is not easy]*

EL7_Se opone al uso de prácticas poco éticas para mejorar el desempeño. *[Opposes the use of unethical practices to improve performance]*

EL8_Considera que la honestidad y la integridad son valores personales importantes. *[Considers honesty and integrity to be important personal values]*

EL9_Comunica normas éticas claras a los trabajadores. *[Communicates clear ethical standards for workers]*

**Felicidad en el trabajo - Workplace Happiness (WH)**

*Factores Relacionados con el puesto de Trabajo - Factors Related to the Job (FRJ)*

FRJ1_En el trabajo obtengo recompensas justas. *[At work I receive fair rewards]*

FRJ2_El clima organizacional de la empresa es bueno. *[The company's organizational climate is good]*

FRJ3_Los jefes dirigen bien. *[The bosses manage well]*

FRJ4_El clima organizacional en mi unidad de trabajo es bueno. *[Organizational climate in my work unit is good]*

FRJ5_La motivación interna por mi puesto es alta. *[Internal motivation for my position is high]*

FRJ6_Mis responsabilidades en la empresa están bien definidas. *[My tasks at the company are well designed]*

FRJ7_Disfruto de mi trabajo. *[I enjoy my work]*

*Factores Relacionados con el Trabajador - Factors Related to the Worker (FRW)*

FRW8_Tengo estabilidad interior. *[I have inner stability]*

FRW9_Tengo bienestar objetivo. *[I have objective well-being]*

FRW10_Tengo estabilidad profesional. *[I have professional stability]*

FRW11_Disfruto haciendo bien mi trabajo. *[I enjoy doing my job well]*

**Identidad Moral del Empleado - Employee Moral Identity (EMI)**

Imagine a una persona ética (caracterizada por ser compasiva, amigable, servicial, generosa, sincera, amable, trabajadora, justa y solidaria). En función a esta premisa, valore las siguientes afirmaciones… *[Imagine an ethical person (characterized as compassionate, friendly, helpful, generous, sincere, kind, hardworking, fair, and supportive). Based on this premise, evaluate the following statements…]*

*Internalización de la Identidad Moral - Moral Identity Internalization (MII)*

MII1_Me haría sentir bien ser una persona con estas características. *[It would make me feel good to be a person who has these characteristics]*

MII2_Ser alguien que tiene estas características es una parte importante de lo que soy. *[Being someone who has these characteristics is an important part of who I am]*

MII3_Deseo fuertemente tener estas características. *[I strongly wish to have these features]*

MII4_Me daría vergüenza ser una persona con estas características. *[I would be ashamed to be a person with these characteristics]*

MII5_Tener estas características no es realmente importante para mí. *[Having these characteristics isn't really important to me]*

*Simbolización de la Identidad Moral - Moral Identity Symbolization (MIS)*

MIS6_Participo activamente en actividades que comunican a otros que tengo estas características. *[I actively participate in activities that communicate to others that I have these characteristics]*

MIS7_A menudo uso ropa que me identifica con estas características. *[I often wear clothes that identify me with these characteristics]*

MIS8_Las actividades que realizo en mi tiempo libre (ej., pasatiempos) me identifican claramente con estas características. *[The kind of things I do in my free time (e.g., hobbies) clearly identify me with these characteristics]*

MIS9_Los libros y revistas que leo me identifican con estas características. *[The types of books and magazines I read identify me with these characteristics]*

MIS10_El hecho de tener estas características se comunica a otros a través de mi membresía en ciertas organizaciones. *[The fact that I have these characteristics is communicated to others through my membership in certain organizations]*

**Clima Ético - Ethical Climate (EC)**

EC1_Este equipo cuenta con un código de ética formal y por escrito. *[This team has a formal, written code of ethics]*

EC2_Este equipo hace cumplir estrictamente un código de conducta. *[This team strictly enforces a code of conduct]*

EC3_Este equipo cuenta con políticas sobre el comportamiento ético. *[This team has policies regarding ethical behavior]*

EC4_Este equipo hace cumplir estrictamente las políticas relacionadas con el comportamiento ético. *[This team strictly enforces policies regarding ethical behavior]*

EC5_El líder de este equipo ha hecho saber con mucha claridad que no se tolerarán comportamientos poco éticos. *[The leader of this team has made it very clear that unethical behavior will not be tolerated]*

**Compromiso Afectivo - Affective Commitment (AC)**

AC1_Me siento emocionalmente conectado con mi equipo de trabajo. *[I feel emotionally connected to my work team]*

AC2_Tengo un fuerte sentido de pertenencia a mi equipo de trabajo. *[I have a strong sense of belonging to my work team]*

AC3_Siento los problemas de mi equipo de trabajo como si fueran los míos. *[I feel as if my team's problems are my own]*

AC4_Mi equipo de trabajo tiene un gran significado personal para mí. *[My work team has great personal significance for me]*

**Voluntad a Reportar Problemas Éticos - Willingness to Report Ethical Problems (WREP)**

WREP1_Me siento cómodo(a) al informar sobre problemas éticos a la gerencia superior. *[I feel comfortable reporting ethical issues to senior management]*
